# Prevalence and predictors of germline *BRCA1* and *BRCA2* mutations among young patients with breast cancer in Jordan

**DOI:** 10.1038/s41598-021-94403-1

**Published:** 2021-07-21

**Authors:** Hikmat Abdel-Razeq, Lama Abujamous, Mahmoud Abunasser, Sara Edaily, Rayan Bater

**Affiliations:** 1 Department of Internal Medicine, King Hussein Cancer Center, Queen Rania Al Abdullah Street, P.O. Box: 1269, Amman, 11941 Jordan; 2 School of Medicine, University of Jordan, Amman, Jordan; 3 Department of Cell Therapy & Applied Genomic, King Hussein Cancer Center, Amman, Jordan

**Keywords:** Health care, Medical research, Oncology, Risk factors

## Abstract

*BRCA1* and *BRCA2* mutations are not uncommon in breast cancer patients. Western studies show that such mutations are more prevalent among younger patients. This study evaluates the prevalence of germline mutations in *BRCA1* and *BRCA2* among breast cancer patients diagnosed at age 40 or younger in Jordan. Blood samples of patients with breast cancer diagnosed at age 40 years or younger were obtained for DNA extraction and *BRCA* sequencing. Mutations were classified as benign/likely benign (non-carrier), pathogenic/likely pathogenic variant (carrier) and variant of uncertain significance (VUS). Genetic testing and counseling were completed on 616 eligible patients. Among the whole group, 75 (12.2%) had pathogenic or likely pathogenic variants; two of the *BRCA2* mutations were novel. In multivariate analysis, triple-negative disease (Odd Ratio [OR]: 5.37; 95% CI 2.88–10.02, P < 0.0001), breast cancer in ≥ 2 family members (OR: 4.44; 95% CI 2.52–7.84, P < 0.0001), and a personal history ≥ 2 primary breast cancers (OR: 3.43; 95% CI 1.62–7.24, P = 0.001) were associated with higher mutation rates. In conclusion, among young Jordanian patients with breast cancer, mutation rates are significantly higher in patients with triple-negative disease, personal history of breast cancer and those with two or more close relatives with breast cancer.

## Introduction

Breast cancer is the most common cancer worldwide and accounts for almost 20% of all cancer cases diagnosed in developing and developed countries^1,2^.A total of 1145 cases were reported by the Jordan Cancer Registry (JCR) in its latest annual report^3^. Similar to many low- and middle-income countries^4^, the median age at breast cancer diagnosis in Jordan is only 52 years, which is ten years younger than most Western societies^5,6^. Additionally, more than a third of patients present with locally-advanced or metastatic disease^7,8^.

Though most breast cancer cases are sporadic, 5–10% of cases are hereditary and mostly related to *BRCA1* or *BRCA2* gene mutations^9^. However, with the widespread use of genetic testing, mutations other than *BRCA1* and *BRCA2* are currently detected. Such mutations include *ATM, CDH1, CHEK2, PALB2, PTEN, STK11*, and *TP53*^10–12^.

Studies had shown that both *BRCA1* and *BRCA2* mutations are associated with a high penetrance rate. The cumulative risk estimates for developing breast cancer by age 80 are 70–90% for carriers of *BRCA1* pathogenic variants and 60–70% for *BRCA2* carriers. The cumulative risk for developing ovarian cancer is a little lower; 40–50% for *BRCA1* carriers and around 20% for *BRCA2* carriers^13,14^. Additionally, the risk of contralateral breast cancer, 20 years after the initial diagnosis, is 40% and 26% for *BRCA1* and *BRCA2* mutation carriers, respectively^15^.

Because of this high penetrance rate and its associated significant consequences, identifying such mutations should be actively sought in high-risk patients identified by international guidelines^13^. Risk-reduction interventions, like bilateral mastectomies and salpingo-oophorectomies, are highly recommended for patients with *BRCA1* or *BRCA2* pathogenic variant carriers, especially so among younger patients.

In addition to its value in preventing breast and ovarian cancers, identification of mutation carriers may have therapeutic importance in patients with breast cancer, too. Recent data had suggested that patients with advanced-stage breast cancer associated with *BRCA1* or *BRCA2* mutations may benefit from PARP (poly ADP ribose polymerase) inhibitors like olaparib and talazoparib; both are currently approved for such situation^16–18^.

Data related to hereditary breast cancer among Arabs, particularly Jordanians, is scarce. Reported pathogenic variant carrier rates vary^19–22^. It is unknown if inherited germline mutations account for earlier age at breast cancer diagnosis in our region. We recently reported our experience on 517 high risk patients treated and followed at our institution; a total of 72 (13.9%) patients had pathogenic or likely pathogenic *BRCA1* or *BRCA2* mutations, while 53 (10.3%) others had a variant of uncertain significance (VUS)^23^.

The diagnosis of breast cancer in young women and its possible genetic implications have potentially serious consequences for patients and their family members, too. Physicians and genetic counselors can help navigate such complex medical and psychosocial issues. In this paper, we aim to study the prevalence and pattern of germline *BRCA1* and *BRCA2* mutations among a group of young Jordanian patients with breast cancer thought to be at higher risk for such mutations.

## Methods

Jordanian breast cancer patients aged 40 years or younger at the time of diagnosis were invited for *BRCA1* and *BRCA2* testing as part of our clinical practice guidelines. Family history or personal history of breast, or other cancers, were not mandated for eligibility. All patients had their diagnosis, treatment, and follow-up at our center.

Eligible patients were identified at their first encounter by a medical oncologist or following the weekly breast multidisciplinary team meetings. Eligible patients who consented to be tested were then referred to a specialized genetic counseling clinic where all potential psychosocial and clinical consequences of positive test results were discussed.

As recommended by international guidelines^15^, *BRCA1* and *BRCA2* variants were classified as benign/likely benign (non-carrier), pathogenic/likely pathogenic (carrier) and VUS. Clinical details and pathological characteristics of the tumors were reviewed. Additionally, a detailed 3-generation family history was also obtained. Estrogen (ER) or progesterone receptors (PR) were positive if tumor cell nuclei staining is ≥ 1%. Human epidermal growth factor receptor-2 (HER-2) was tested using a standardized immune histochemical staining (IHC), and tumor cells were considered negative with scores of 0 or + 1, and positive for those with + 3 scores. Fluorescence in situ hybridization (FISH) was performed for equivocal samples with + 2 scores. Triple-negative tumors are those which tested negative for ER, PR, and HER-2.

Blood samples were obtained for DNA extraction, full-gene sequencing, and deletion/duplication analysis for *BRCA1* and *BRCA2* using next-generation sequencing technology (NGS) and/or Multiplex Ligation-dependent Probe Amplification (MLPA) analysis were performed at three reference labs: Myriad Genetics laboratory (Salt Lake City, UT), Leeds Cancer Center (Leeds, United Kingdom) and invitae (San Francisco, CA).

Our study was carried out in accordance with the code of ethics of the World Medical Association (Declaration of Helsinki) and was approved by the Institutional Review Board (IRB) at King Hussein Cancer Center. All patients signed informed consent.

### Statistical analysis

Patients’ clinical and pathologic characteristics were collected, tabulated, and described by ranges, medians, or percentages. Relatives diagnosed with breast cancer and tested after the family’s index case were not enrolled and were excluded from the analysis. Chi-square tests were used to compare the proportion of *BRCA1* and *BRCA2* pathogenic/likely pathogenic variant carriers according to age (≤ 30 versus > 30), triple-negative status, and family history. Multivariate analysis using a logistic regression model was performed. Odds ratios and their related 95% confidence intervals (CI) were calculated. P-value ≤ 0.05 was considered significant. Analyses were conducted using Minitab Statistical Software version 18 (Minitab 18 Statistical Software (2017). State College, PA: Minitab, Inc. (www.minitab.com).

### Ethics approval and consent to participate

The study was approved by King Hussein Cancer Center’s Institutional Review Board (IRB). All patients signed informed consent.

### Consent for publication

Data submitted are entirely unidentifiable and there are no details on individuals reported within the manuscript. Request to publish was approved by King Hussein Cancer Center IRB.

## Results

Between November 2016 and January 2020, 616 eligible patients were recruited. Participants’ median age was 35 (range 19–40) years, and 121 (19.6%) patients were 30 years or younger. The majority (n = 482, 78.2%) of the patients had hormone receptor (ER and/or PR) positive disease. HER-2 testing was available on 547 patients, 180 (32.9%) were positive by IHC and/or FISH, and 69 (12.6%) had triple-negative disease Table 1.

**Table 1:** Patients Characteristics (n = 616).

Characteristics		Number	Percentage (%)
Age at diagnosis (years)	Median	35	
	Range	19–40	
Hormonal status	ER-positive	449	73.0
	PR-positive	438	71.0
	ER and/or PR-positive	482	78.2
	ER and PR-negative	134	22.0
HER-2 status*	HER2-positive	180	32.9
	HER2-negative	367	67.1
	Unknown	68	12.4
Triple negative*		69	12.6
Positive family history of breast cancer		499	81.0

*Percentage from 547 with known HER2 status.

*ER* estrogen receptors, *PR* progesterone receptors, *HER2* human epidermal growth factor receptor-2.

Among the whole group, 75 (12.2%) patients had pathogenic/likely pathogenic *BRCA1* or *BRCA2* mutations; 50 (66.7%) were in *BRCA2*, while an additional 57 (9.3%) had a VUS (Supplementary Table S1). Patients with at least two breast cancer primaries (n = 48) had a significantly high mutation rate (n = 8, 29.2%). Table 2 presents mutation rates according to different categories.

**Table 2:** Rates of positive *BRCA1* and *BRCA2* mutations; subgroup analysis.

Variable		Total	Positive Mutations			
			*BRCA1*	*BRCA2*	*BRCA1*& *BRCA2*	P-Value*
Age at diagnosis (years)	≤ 35	341	16	34	50 (14.7%)	0.017
	> 35	275	9	16	25 (9.1%)	
One or more close relative with breast cancer at any age	Yes	305	9	37	46 (15.1%)	0.029
	No	311	15	14	29 (9.3%)	
One or more close relatives with breast cancer diagnosed at age 50 years or younger	Yes	153	3	24	27 (17.6%)	0.017
	No	463	22	26	48 (10.4%)	
Diagnosed at ≤ 60 years with triple negative disease	Yes	69	16	7	23 (33.3%)	< 0.001
	No	547	9	43	52 (9.5%)	
Any age with at least 2 breast cancer primaries	Yes	48	6	8	14 (29.2%)	< 0.001
	No	568	19	42	61 (10.7%)	
Two or more close relatives with breast cancer	Yes	97	5	24	29 (30.0%)	< 0.001
	No	519	20	26	46 (8.9%)	
All patients		616	25	50	75 (12.2%)	

### Family history

**Figure 1:**
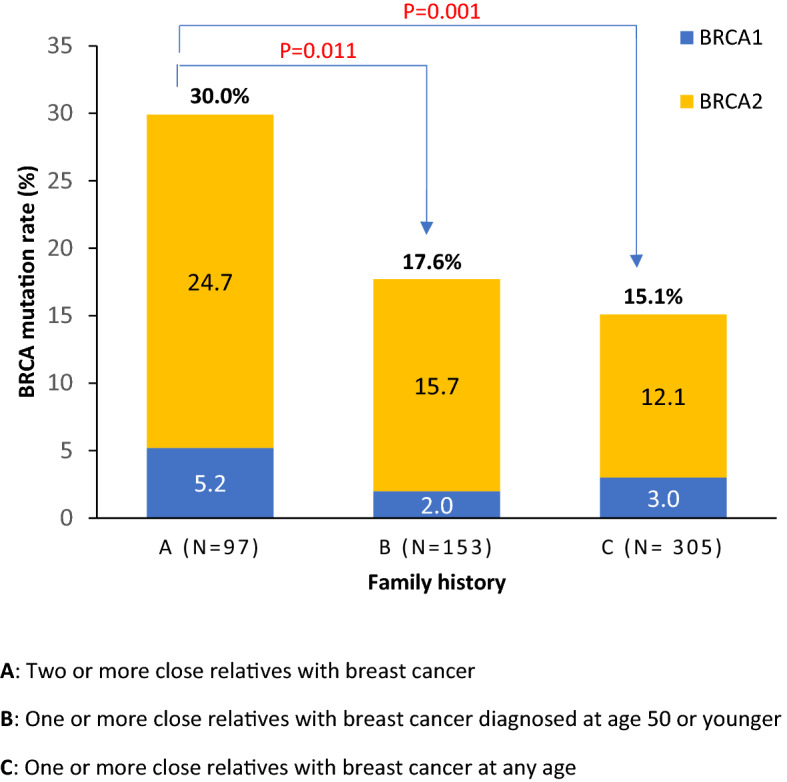
*BRCA1* and *BRCA2* mutation rates by family history. (**A**) Two or more close relatives with breast cancer. (**B**) One or more close relatives with breast cancer diagnosed at age 50 years or younger. (**C**) One or more close relatives with breast cancer at any age.

The majority of the patients enrolled (n = 499, 81.0%) had a positive family history of breast cancer in first-, second- or third-degree family members. Women with *two or more* close relatives diagnosed at any age with breast cancer (Group-A, n = 97) had the highest mutation rate (n = 29, 30.0%). In contrast, women with *one or more* family members diagnosed with breast cancer before the age of 50 years (Group-B, n = 153) had a mutation rate of 17.6%, P = 0.011. The mutation rate was lower (15.1%, P = 0.001) among women with one or more family members diagnosed at any age (Group-C, n = 305), Fig. 1.

### Age at diagnosis

**Figure 2:**
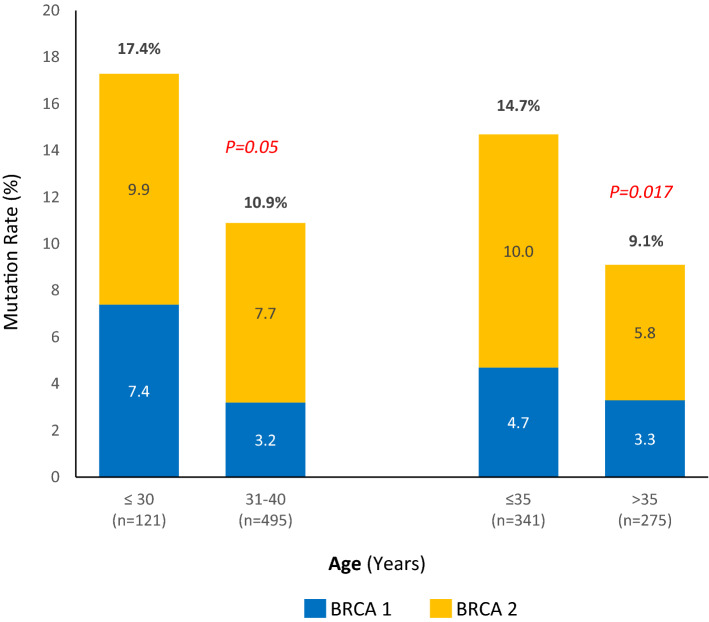
*BRCA1* and *BRCA2* mutation rates by age group.

We studied the contribution of age to mutation rate in two ways. First, we compared mutation rates across the median age of our cohort; *BRCA1* or *BRCA2* pathogenic variants were reported in 14.7% of 341 patients aged ≤ 35 years, compared to 9.1% in 275 patients older than 35 years, P = 0.017. Second, we compared mutation rates across two age groups: < 30 years and those aged 31–40 years; mutation rates were 17.4% and 10.9% (P = 0.05), respectively, Fig. 2.

### Triple-negative disease

Patients with triple-negative disease (n = 69) had significantly higher rates (n = 23, 33.3%) compared to 9.5% among non-triple negative patients, P < 0.001. Most of the pathogenic variants were in *BRCA1* (n = 16, 23.2%), and the majority (n = 54, 78.3%) of such patients had a positive family history of breast cancer; only 2 (13.3%) of the 15 patients with no family history had a pathogenic variant.

### Multivariate analysis

In the multivariate analysis, triple-negative disease (Odds Ratio [OR]: 5.37; 95% CI 2.88–10.02, P < 0.0001), breast cancer in two or more family members (OR: 4.44; 95% CI 2.52–7.84, P < 0.0001), and a personal history of two or more primary breast cancer (OR: 3.43; 95% CI 1.62–7.24, P = 0.001), were associated with higher *BRCA* mutation rates.

### Mutation types

A spectrum of 39 different mutations, 22 in *BRCA2* and 17 in *BRCA1*, were detected (Tables 3 and 4). To our knowledge, two mutations in *BRCA2* (c.6193C > T in exon 11 and c.1013del in exon 10) have not been reported previously in any database. Additionally, five unrelated females in our cohort were found to harbor two concomitant mutations in *BRCA2* exon11 (c.2254_2257del) and (c.5351dup), simultaneously (Table 4). These two mutations appeared separately in a very limited number of studies^24–26^. Except for mutations c.1233dup and c.9257-1G>A/IVS24-1G>A, for which two family members were tested for each, all other variants have been detected in different families. Nineteen (25.3%) of the mutations detected in our patients were either (c.2254_2257del) or (exon 5–11 duplication); both in *BRCA2* gene and were detected in 11 and 8 different patients, respectively.

**Table 3:** Types of *BRCA1* mutations.

Gene	Exon/intron	Nucleotide change	Amino acid change	Variant type	Database report	Frequency
BRCA 1	Exon 1–2	Deletion (exons 1–2)	Absent or disrupted protein product	Large deletion	Yes	1
BRCA 1	Exon 2	c.66dup	p.Glu23Argfs	Duplication/fs	Yes	1
BRCA 1	Exon 3	c.121C > T	p.His41Tyr	Missense	Yes	1
BRCA 1	Exon 10	c.3835del	p.Ala1279Hisfs	Deletion/fs	Yes	1
BRCA 1	Exon 11	c.3436_3439del	p.Cys1146LeufsTer	Deletion/fs	Yes	2
BRCA 1	Exon 11	c.798_799del	p.Ser267Lysfs	Deletion/fs	Yes	1
BRCA 1	Exon 11	c.2761C > T	p.Gln921Ter	Nonsense	Yes	1
BRCA 1	Exon 11	c.1961del	p.Lys654Serfs	Deletion/fs	Yes	1
BRCA 1	Exon 11	c.809del	p.His270Leufs	Deletion/ fs	Yes	1
BRCA 1	Exon 11	c.4065_4068del	p.Asn1355Lysfs	Deletion/fs	Yes	2
BRCA 1	Exon 12	c.4117G > T	p.Glu1373Ter	Nonsense	Yes	4
BRCA 1	Exon 15	c.4524G > A	p.Trp1508Ter	Nonsense	Yes	1
BRCA 1	Exon 17	c.5030_5033del	p.Thr1677Ilefs	Deletion/fs	Yes	1
BRCA 1	Exon 18	c.5123C > A	p.Ala1708Glu	Missense	Yes	2
BRCA 1	Exon 18	c.5095C > T	p.Arg1699Trp	Missense	Yes	1
BRCA 1	Exon 19	c.5161C > T	p.Gln1721Ter	Nonsense	Yes	1
BRCA 1	Intron 17	c.5074 + 3A > G/ IVS17 + 3	Splice acceptor	Intervening sequence	Yes	3

**Table 4:** Types of *BRCA2* mutations.

Gene	Exon/intron	Nucleotide change	Amino acid change	Variant type	Database report	Frequency
BRCA 2	Exons 5–11	exon 5–11 duplication	Absent or disrupted protein product	Large duplication	Yes	8
BRCA 2	Exon 8	c.658_659del	p.Val220Ilefs	Deletion/fs	Yes	1
BRCA 2	Exon 10	c.1233dup	Pro412Thrfs	Duplication/fs	Yes	5
BRCA 2	Exon 10	c.1013del	p.Ala338Metfs	Deletion/fs	No	1
BRCA 2	Exon 11	c.2254_2257del	p.Asp752Phefs	Deletion/fs	Yes	11
BRCA 2	Exon11/ Exon11	c.2254_2257del & c.5351dup	p.Asp752Phefs & p.Asn1784Lysfs	Deletion/fs-Duplication/fs	No	5
BRCA 2	Exon 11	c.6685G > T	p.Glu2229Ter	Nonsense	Yes	3
BRCA 2	Exon 11	c.6486_6489del	p.Lys2162Asnfs	Deletion/fs	Yes	2
BRCA 2	Exon 11	c.4222_4223del	p.Gln1408Argfs	Deletion/fs	No	2
BRCA 2	Exon 11	c.6627_6634del	p.Ile2209Metfs	Deletion/fs	Yes	2
BRCA 2	Exon 11	c.2677C > T	p.Gln893Ter	Nonsense	Yes	1
BRCA 2	Exon 11	c.6193C > T	p.Gln2065Ter	Nonsense	No	1
BRCA 2	Exon 11	c.2808_2811del	p.Ala938Profs	Deletion/fs	Yes	1
BRCA 2	Exon 11	c.4936_4939del	p.Glu164Gln6fs	Deletion/fs	Yes	1
BRCA 2	Exon 11	c.5722_5723del	p.Leu1908Argfs	Deletion/fs	Yes	1
BRCA 2	Exon 11	c.6445_6446del	p.Ile2149Ter	Deletion/fs	Yes	1
BRCA 2	Exon 11	c.6022A > T	p.Lys2008Ter	Missense	Yes	1
BRCA 2	Exon 13	c.7007G > A	p.Arg2336His	Missense	Yes	1
BRCA 2	Exon 18	c.8140C > T	p.Gln2714Ter	Nonsense	Yes	1
BRCA 2	Exon 22	c.8878C > T	p.Gln2960Ter	Nonsense	Yes	2
BRCA 2	Exon 22	c.8760 T > G	p.Tyr2920Ter	Nonsense	Yes	1
BRCA 2	Intron 24	c.9257-1G > A/ IVS24-1G > A	Splice acceptor	Intervening sequence	Yes	3

## Discussion

Our study confirms that younger patients are at a higher risk of harboring pathogenic or likely pathogenic mutations and such risk is higher for patients younger than 30 years at the time of breast cancer diagnosis. However, differences in mutation rates between patients above or below 40 years is less obvious. In one of our previous studies, the mutation rate among 333 younger patients (≤ 40 years) was 13.2% compared to 15.2% among 184 older ones, P = 0.53^23^.

Our findings of two novel mutations that have been detected in our database as well as a higher frequency of certain mutations like (c.2254_2257del) and (exon 5–11 duplication) will probably have an important consequence for the genetic testing of *BRCA* genes in Jordan where consanguineous marriage is relatively common. In one study, researchers reviewed published and unpublished data to identify population‐specific founder *BRCA* pathogenic sequence variants (PSVs) in Middle East, North Africa, and Southern Europe; 232 PSVs in *BRCA1* and 239 in *BRCA2* were identified^27^.

It is also worth highlighting that our study identifies three risk factors, the presence of any of which in younger patients increases the pathogenic variant carrier rate to almost one in three tested patients. These include, patients with triple-negative disease, women with at least two breast primaries, and those with a family history of breast cancer in two or more close relatives diagnosed at any age. Such findings might help simplify our efforts to educate both patients and health care providers about the importance of genetic testing and counseling for such patients.

Our VUS rate (9.3%) is higher than what had been reported among Caucasian patients^28^. This rate will probably go even higher with the wider implementation of multi-gene testing. Several studies had shown higher VUS rates among African-Americans, Hispanics and patients of Ashkenazi–Jewish descent^29–31^.

We have built a good experience in dealing with patients before and after testing. Ensuring confidentiality was never a problem in our current daily practice. Very few patients refused genetic testing and counseling because of their fear of stigmatization and labeling. However, prophylactic bilateral mastectomies and oophorectomies with reconstructive surgery can be a challenge. Studies addressing the psychosocial consequences of pathogenic variants especially among younger patients in our region, are highly needed.

Though our study represents a single-center, we believe it reflects the whole country as our institution treats most of the country’s breast cancer cases. However, our study is not without limitations; issues related to psychosocial aspects related to pathogenic variant carrier state, risk-reduction surgeries, fertility-related issues, and outcome of family members at-risk of index cases need to be followed and addressed.

## Conclusions

*BRCA1* and *BRCA2* mutation rates among patients 40 years or younger are relatively high but not necessarily higher than older patients. However, personal and family risk factors can identify subgroups of younger patients with much higher mutation rates.

## Supplementary Information

Supplementary Information.

## Data Availability

Data will not be available online as it might contain sensitive information. Data will be available through the corresponding author on reasonable requests.

## Abbreviations

CI: Confidence intervals
ER: Estrogen receptors
FISH: Fluorescent in situ hybridization
HER2: Human epidermal growth factor receptor
IHC: Immunohistochemistry
IRB: Institutional review board
MLPA: Multiplex ligation-dependent probe amplification
NCCN: National comprehensive cancer network
NGS: Next-generation sequencing
OR: Odds ratio
PR: Progesterone receptors
PARP: Poly ADP ribose polymerase
VUS: Variant of uncertain significance
