# Supplementary material for: Prevalence and predictors of germline BRCA1 and BRCA2 mutations among young patients with breast cancer in Jordan
Source: Sci Rep. 2021 Jul 21;11:14906. doi: 10.1038/s41598-021-94403-1 (PMC8295261; doi:10.1038/s41598-021-94403-1)
Supplement: Supplementary file 1 — Supplementary Information. [file 41598_2021_94403_MOESM1_ESM.docx]

**Table-1S** (Supplementary): Variants of Uncertain Significance (VUS)

| Gene | Nucleotide change | Amino acid change | Variant type | Interpretation of pathogenicity​ by ClinVar |
| --- | --- | --- | --- | --- |
| BRCA1 | c.1446_1448del | p.Ile483del | Deletion | VUS |
| BRCA1 | c.2123C>A | p.Ser708Tyr | Missense | Conflicting interpretations  Likely benign; Uncertain significance |
| BRCA1 | c.2604A>G | p.Ser868= | Synonymous | Likely benign​ |
| BRCA1 | c.3149G>C | p.Ser1050Thr | Missense | VUS |
| BRCA1 | c.3526 G>A | p.Val1176Ile | Missense | VUS |
| BRCA1 | c.3587C>T | p.Thr1196Ile | Missense | VUS |
| BRCA1 | c.3642G>T | p.Glu1214Asp | Missense | VUS |
| BRCA1 | c.4028A>T | p.Asp1343Val | Missense | VUS |
| BRCA1 | c.4185+10G>A | NA | Intron variant | Conflicting interpretations Benign; Likely benign; Uncertain significance |
| BRCA1 | c.4357+5G>A | NA | Intron variant | VUS |
| BRCA1 | c.478G>A | p.Gly160Arg | Missense | VUS |
| BRCA1 | c.5333-6T>C | NA | Intron variant | NA |
| BRCA1 | c.5555C>T | p.Thr1852Ile | Missense | VUS |
| BRCA1 | c.693G>A | p.Thr231= | Synonymous | Likely benign |
| BRCA1 | c.851A>G | p.Gln284Arg | Missense | VUS |
| BRCA1 | c.994C>T | p.Arg332Trp | Missense | Conflicting interpretations Likely benign; Uncertain significance |
| BRCA1 | exons 5-8 duplication | NA | Duplication | upgraded from uncertain clinical significance to likely pathogenic |
| BRCA2 | c.10202C>T | p.Thr3401Met | Missense | Conflicting interpretations Benign; Likely benign; Uncertain significance |
| BRCA2 | c.122C>T | p.Pro41Leu | Missense | Conflicting interpretations Benign; Likely benign; Uncertain significance |
| BRCA2 | c.1550A>G | p.Asn517Ser | Missense | Conflicting interpretations Likely benign; Uncertain significance |
| BRCA2 | c.1769T>G | p.Phe590Cys | Missense | Conflicting interpretations Likely benign; Uncertain significance |
| BRCA2 | c.1793C>T | p.Thr598Ile | Missense | VUS |
| BRCA2 | c.2072 C>T | p.Ala691Val | Missense | VUS |
| BRCA2 | c.2366 A>G | p.Lys789Arg | Missense | VUS |
| BRCA2 | c.2396A>G | p.Lys799Arg | Missense | VUS |
| BRCA2 | c.277T>G | p.Ser93Ala | Missense | VUS |
| BRCA2 | c.280 C>T | p.Pro94Ser | Missense | Conflicting interpretations Likely benign; Uncertain significance |
| BRCA2 | c.3676A>C | p.Lys1226Gln | Missense | VUS |
| BRCA2 | c.4252A>G | p.Ile1418Val | Missense | VUS |
| BRCA2 | c.4594G>T | p.Val1532Phe | Missense | VUS |
| BRCA2 | c.4621A>C | p.Lys1541Gln | Missense | VUS |
| BRCA2 | c.4943C>T | p.Ala1648Val | Missense | VUS |
| BRCA2 | c.502C>G | p.Pro168Ala | Missense | VUS |
| BRCA2 | c.5126A>C | p.Asp1709Ala | Missense | VUS |
| BRCA2 | c.5590G>A | p.Asp1864Asn | Missense | VUS |
| BRCA2 | c.5897A>G | p.His1966Arg | Missense | Conflicting interpretations Benign; Likely benign; Uncertain significance |
| BRCA2 | c.62A>G | p.Lys21Arg | Missense | Conflicting interpretations Likely benign; Uncertain significance |
| BRCA2 | c.6986 C>T | p.Pro2329Leu | Missense | Conflicting interpretations Likely benign; Uncertain significance |
| BRCA2 | c.7301A>C | p.Lys2434Thr | Missense | VUS |
| BRCA2 | c.752C>G | p.Thr251Arg | Missense | VUS |
| BRCA2 | c.7534C>T | p.Leu2512Phe | Missense | Benign​ |
| BRCA2 | c.7806-6G>T | NA | Intron variant | Likely benign |
| BRCA2 | c.8332-3C>G | NA | Intron variant | VUS |
| BRCA2 | c.8632G>C | p.Glu2878Gln | Missense | VUS |
| BRCA2 | c.865A>G | p.Asn289Asp | Missense | Conflicting interpretations Likely benign; Uncertain significance |
| BRCA2 | c.8755-19 A>G | NA | Intron variant | Conflicting interpretations Likely benign; Uncertain significance |
| BRCA2 | c.9502-12T>G | NA | Intron variant | Benign​ |
| BRCA2 | c.9586A>G | p.Lys3196Glu | Missense | Conflicting interpretations Benign; Likely benign; Uncertain significance |
| BRCA2 | c.9754_9765del | p.Ser3252_Gly3255del | Deletion | VUS |
| BRCA2 | c.9781G>A | p.Asp3261Asn | Missense | VUS |
| BRCA2 | c.9839C>A | p.Pro3280His | Missense | Conflicting interpretations Likely benign; Uncertain significance |
| BRCA2 | c.9875C>T | p.Pro3292Leu | Missense | Benign​ |
